# Supplementary material for: Phylogenetic Analysis and Structural Evaluation of Staphylococcus aureus Serine-Aspartate Repeat-Containing Protein D with a Focus on Periprosthetic Joint Infection
Source: bioRxiv. 2026 May 5:2026.05.01.722179. Preprint. [Version 1] doi: 10.64898/2026.05.01.722179 (PMC13174682; doi:10.64898/2026.05.01.722179)
Supplement: 1 [file NIHPP2026.05.01.722179v1-supplement-1.pdf]

bioRxiv preprint doi: https://doi.org/10.64898/2026.05.01.722179; this version posted May 5, 2026. The copyright holder for this preprint (which was not certified by peer review) is the author/funder, who has granted bioRxiv a license to display the preprint in perpetuity. It is made available under aCC-BY 4.0 International license.

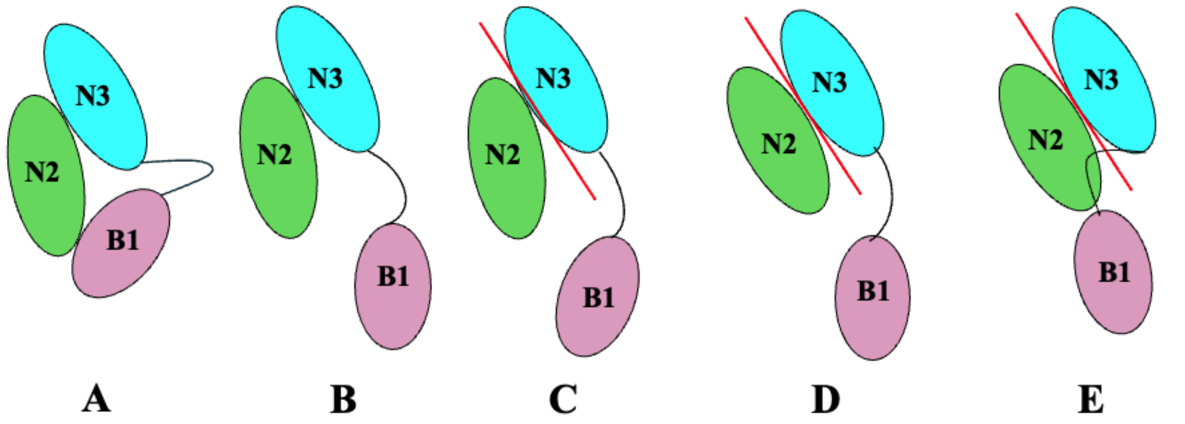

Figure S1. Presumed DLL mechanism for *Staphylococcus aureus* SdrD based on the "dock, lock, and latch (DLL)" ligand-binding mechanism. Only the N2, N3, and B1 domains were used for modeling. (A) The conformation of the N2N3B1 domains observed in crystal structures is regarded an *Apo* state. (B) When the B1 domain detaches from the N2 domain, the conformation is designated an open state. (C) The step in which a peptide segment from a host protein binds in the trench between the N2 and N3 domains, forming an edge β-strand of N3, is referred to as docking. (D) The subsequent locking step represents conformational adjustments within and/or between the N2 and N3 domains that tighten around the bound ligand, stabilizing the complex. (E) The final latching step occurs when the linker peptide between the N3 and B1 domains swings over the bound peptide and inserts into a complementary groove on the N2 domain, forming an additional β-strand. This closes the binding site and yields a highly stable final complex.

bioRxiv preprint doi: https://doi.org/10.64898/2026.05.01.722179; this version posted May 5, 2026. The copyright holder for this preprint (which was not certified by peer review) is the author/funder, who has granted bioRxiv a license to display the preprint in perpetuity. It is made available under aCC-BY 4.0 International license.

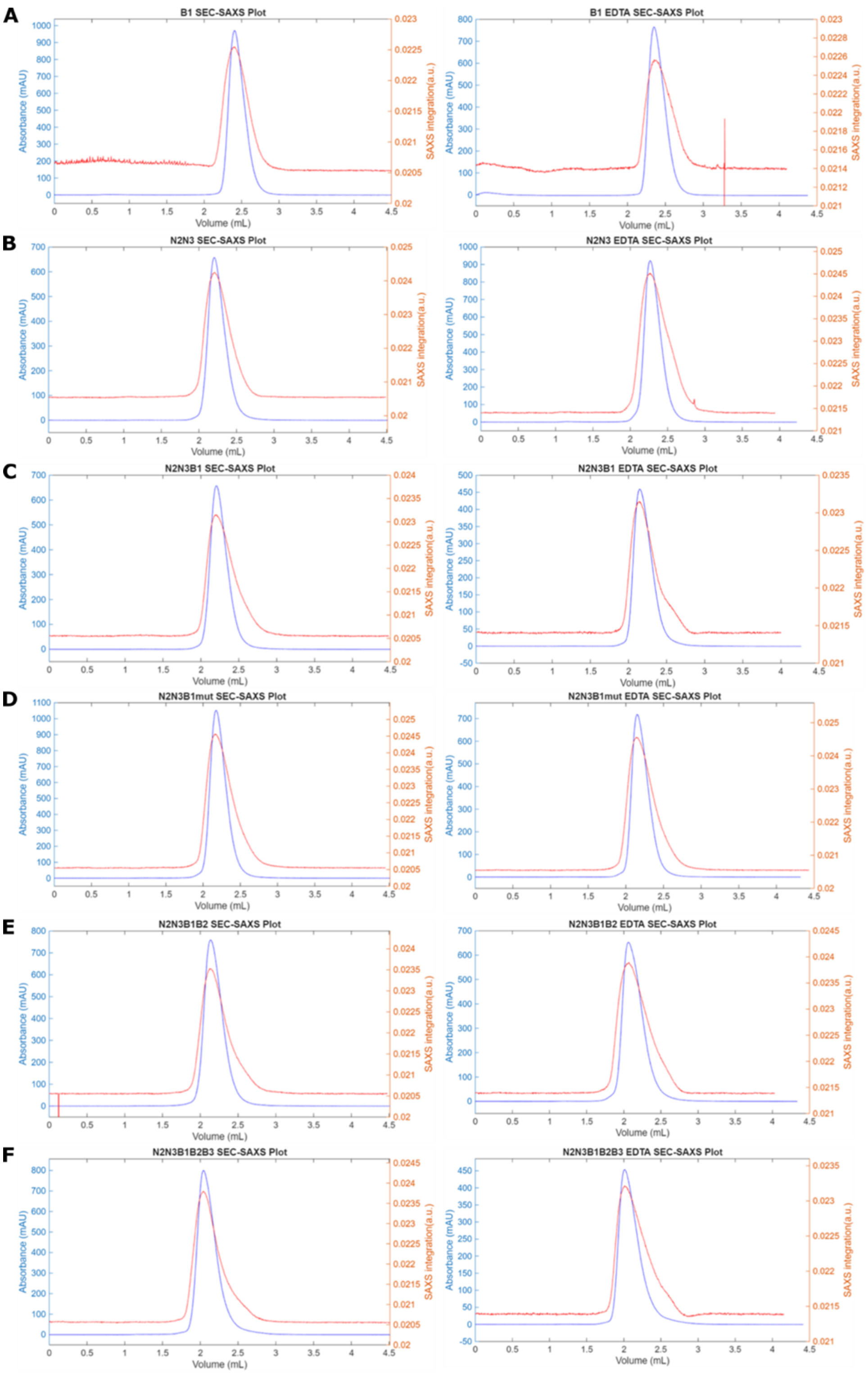

Figure S2. Superimposed SEC profiles detected with UV-vis absorbance at 280 nm (left axis) and SAXS signal integration (right axis) for *Staphylococcus aureus* SdrD. (A) B1, (B) N2N3, (C) N2N3B1, (D) N2N3B1 N574A mutant, (E) N2N3B1B2, (F) N2N3B1B2B3, in protein buffer (20 mM HEPES pH 7.5, 150 mM NaCl, 1 mM TCEP) (left) and in protein buffer with 10 mM EDTA added (right). Profiles detected with SAXS are generally broader than those with UV because SAXS detection is located downstream where the chamber is larger.

bioRxiv preprint doi: https://doi.org/10.64898/2026.05.01.722179; this version posted May 5, 2026. The copyright holder for this preprint (which was not certified by peer review) is the author/funder, who has granted bioRxiv a license to display the preprint in perpetuity. It is made available under aCC-BY 4.0 International license.

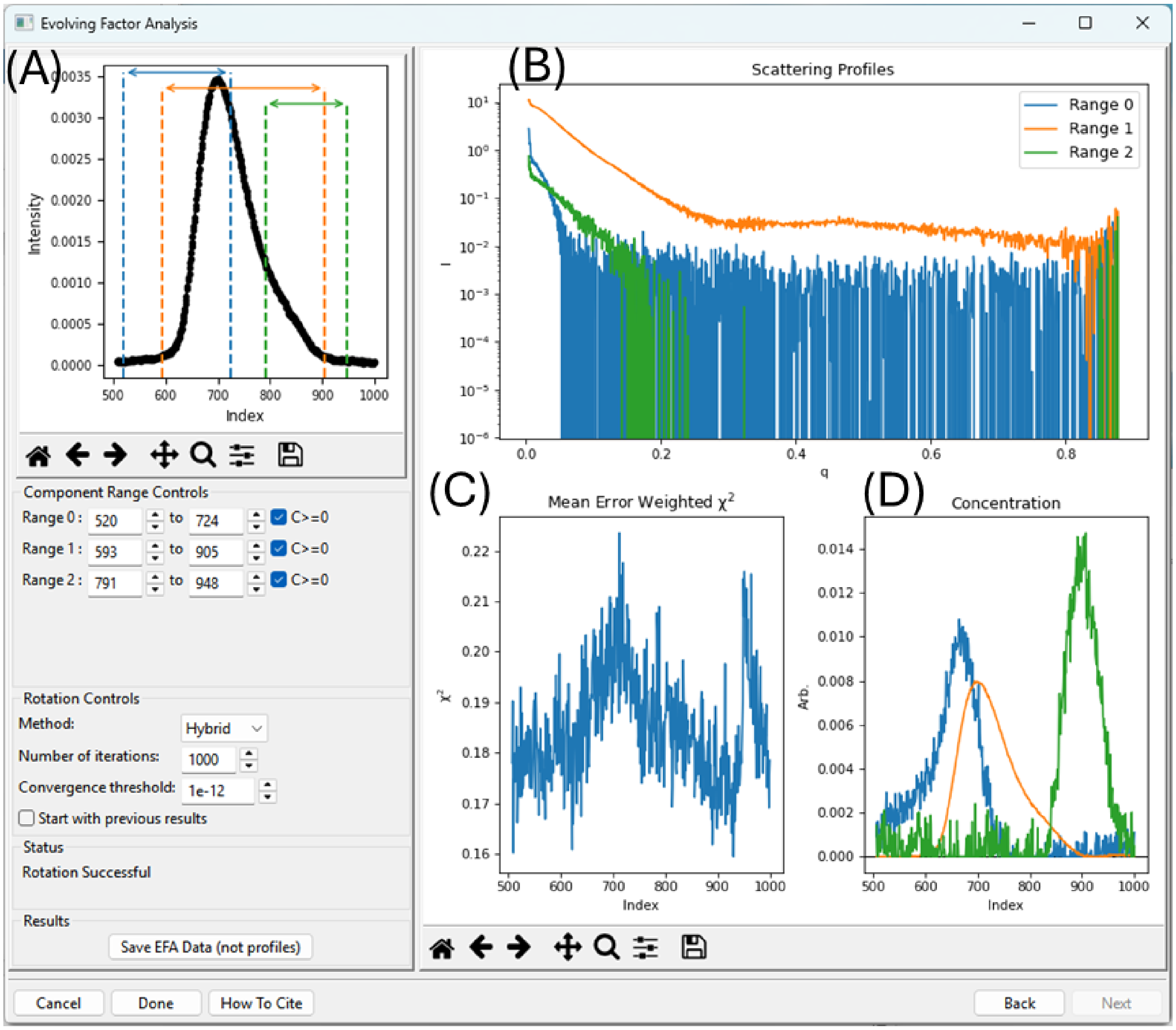

Figure S3. Representative EFA (evolving factor analysis) SEC-SAXS data analysis in BioXTAS RAW. Analysis screenshot captured from RAW for *Staphylococcus aureus* SdrD N2N3B1B2 in protein buffer. (A) Three possible components were identified and their respective existing ranges. (B) The SAXS profiles recovered from EFA analysis for the three components. The second component colored in orange is the major component. (C) Individual fit-goodness plot for SAXS data. (D) Concentration profiles for each component based on SAXS spectra in (B).

bioRxiv preprint doi: https://doi.org/10.64898/2026.05.01.722179; this version posted May 5, 2026. The copyright holder for this preprint (which was not certified by peer review) is the author/funder, who has granted bioRxiv a license to display the preprint in perpetuity. It is made available under aCC-BY 4.0 International license.

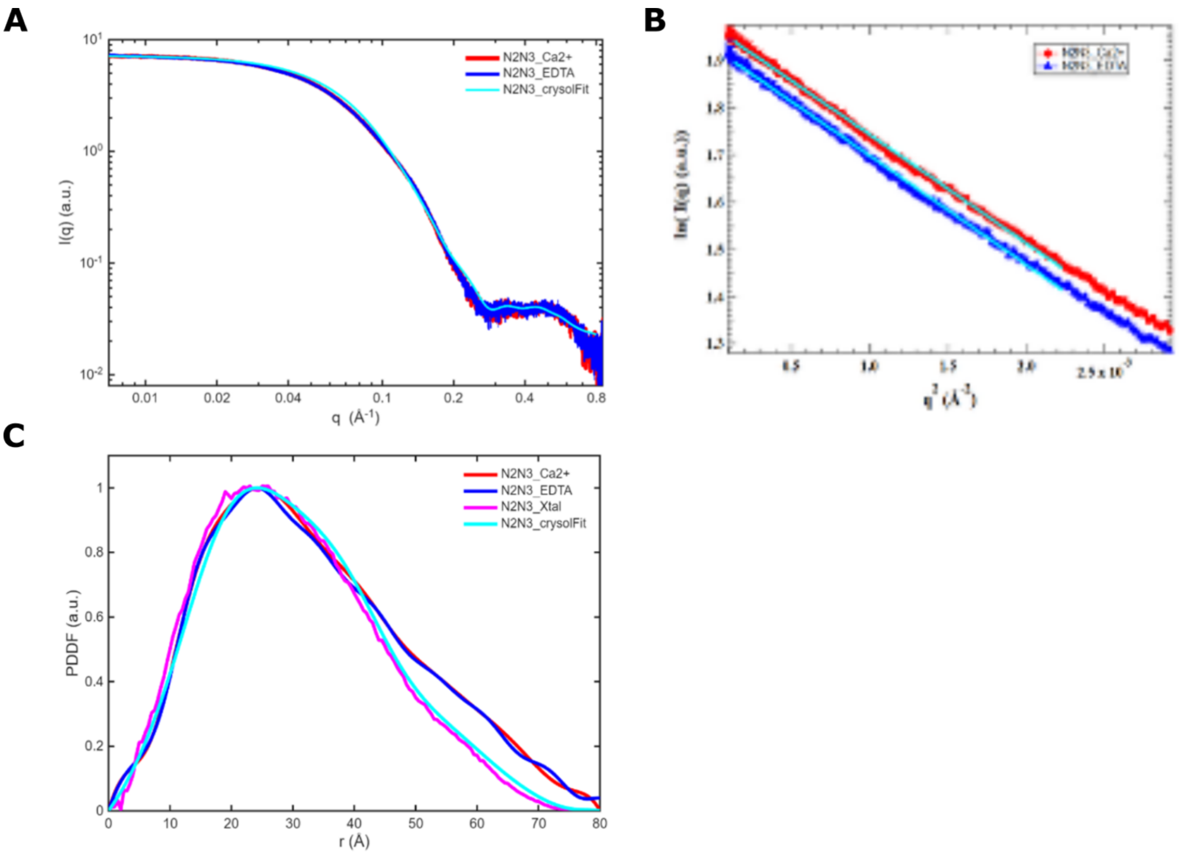

Figure S4. SAXS results for *Staphylococcus aureus* SdrD N2N3 domains. (A) Superimposed SAXS profiles of N2N3 in protein buffer (red curve), and in protein buffer with 10 mM EDTA added (blue). (B) Guinier plots for protein N2N3 in the presence of Ca^{2+} (red) and EDTA (blue), respectively. Cyan lines are Guinier fittings yielding radius of gyration (R_g) values: 26.2±0.1 Å with Ca^{2+}; 26.3±0.2 Å with EDTA. (C) PDDF profiles for N2N3. Red, calculated from experimental SAXS data (red curve in Figure S4A) using GNOM for N2N3 with Ca^{2+}; blue, calculated from SAXS data (blue curve inf Figure 2D) for sample with EDTA. Cyan, calculated from fitting SAXS curve (cyan in S4A) using GNOM. Magenta, PDDF calculated from the crystal structure using program SolX2. The difference between cyan and magenta PDDFs arises from the hydration layer added in the CRYSOL fitting.

bioRxiv preprint doi: https://doi.org/10.64898/2026.05.01.722179; this version posted May 5, 2026. The copyright holder for this preprint (which was not certified by peer review) is the author/funder, who has granted bioRxiv a license to display the preprint in perpetuity. It is made available under aCC-BY 4.0 International license.

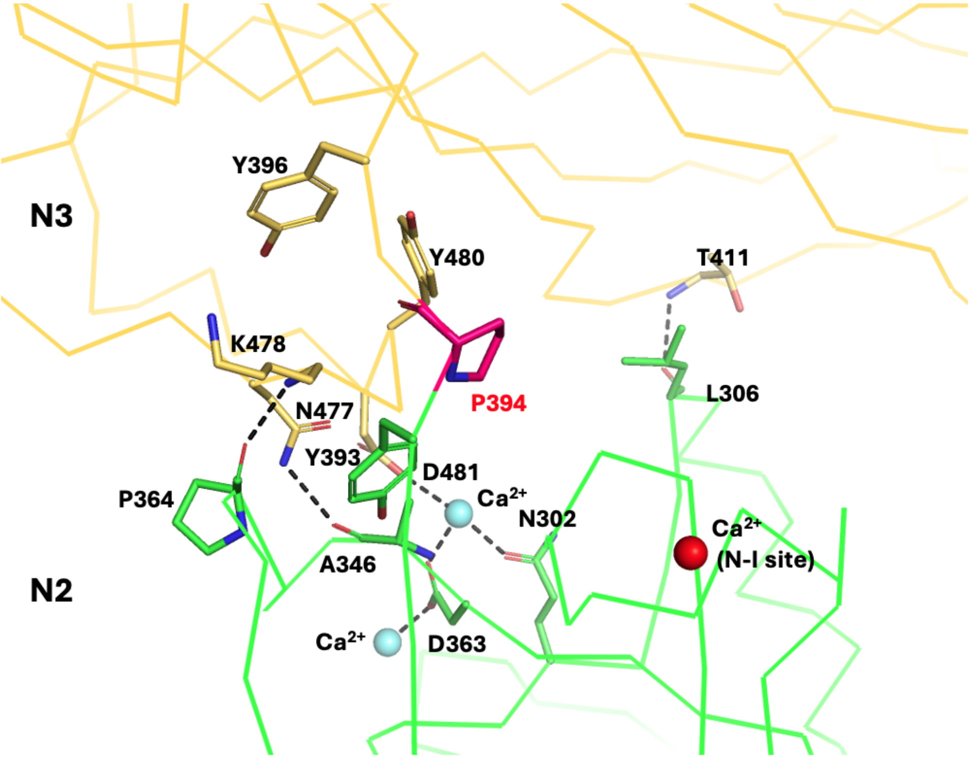

Figure S5. Alpha-trace diagram of the interface between *Staphylococcus aureus* SdrD N2 (green) and N3 (light orange) domains and the primary interactions across their interface. Residues involved in the interactions are shown in stick format. Link residues between the two domains, P394, is highlighted in magenta. Ca^{2+} at the N-I site is shown as a red sphere, while the two Ca^{2+} ions located at N-III site are shown as cyan spheres, see Figure 2A-C. This diagram was prepared based on the *S. aureus* SdrD N2N3 structure.

bioRxiv preprint doi: https://doi.org/10.64898/2026.05.01.722179; this version posted May 5, 2026. The copyright holder for this preprint (which was not certified by peer review) is the author/funder, who has granted bioRxiv a license to display the preprint in perpetuity. It is made available under aCC-BY 4.0 International license.

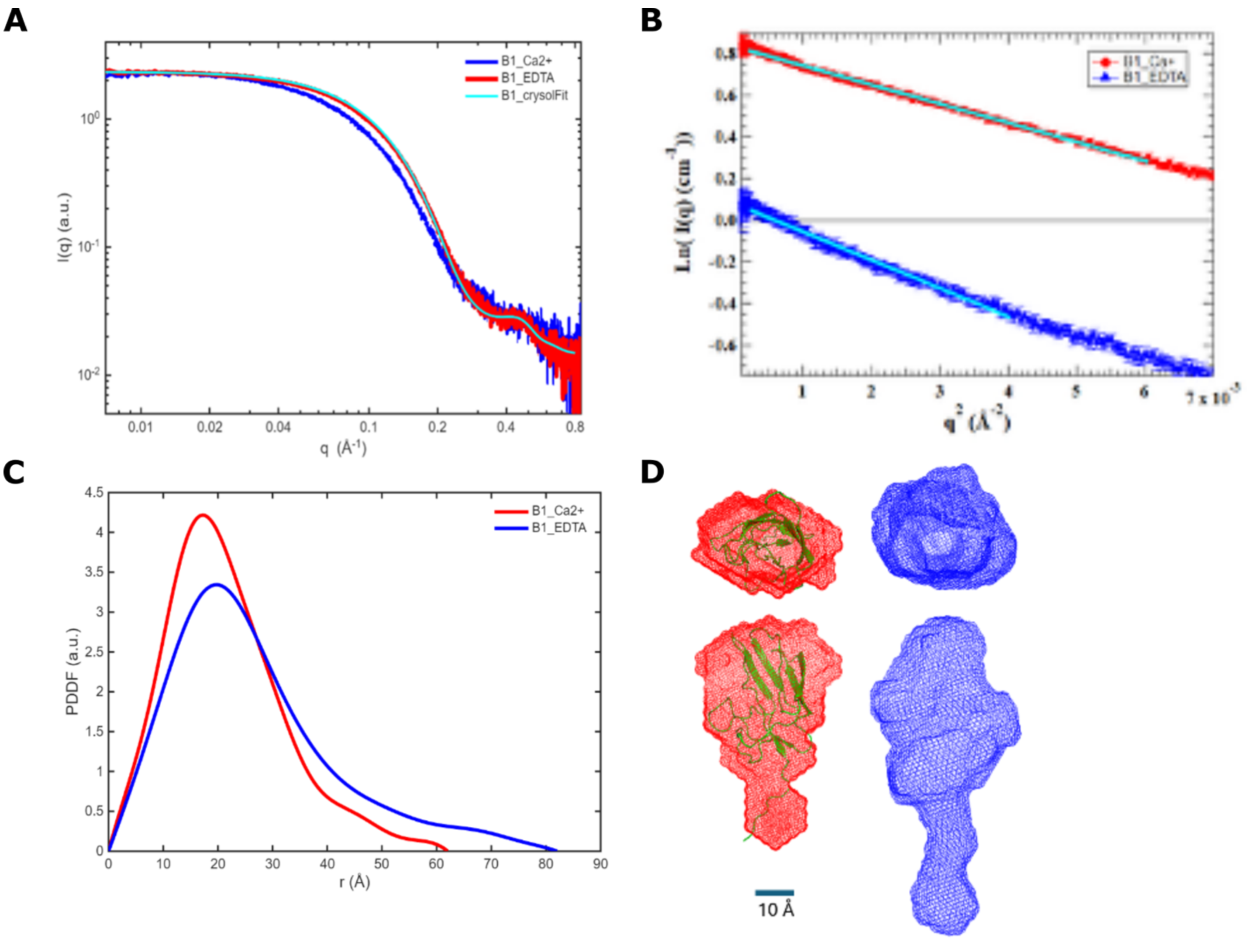

Figure S6. SAXS results for *Staphylococcus aureus* SdrD B1 domain. (A) Superimposed SAXS profiles of B1 in protein buffer (red curve), in protein buffer with 10 mM EDTA added (blue), and fitting with the crystal structure (cyan). The goodness-of-fit (chi2) against the red curve is 0.58. (B) Guinier plots for protein B1 in the presence of Ca^{2+} (red) and EDTA (blue), respectively. Cyan lines are the fitting. Radius of gyration (R_g) values: 16.6±0.1 Å with Ca^{2+}; 20.2±0.2 Å with EDTA. (C) PDDF profiles for N2N3. Red, calculated from experimental SAXS data (red curve in Figure S5A) using GNOM; blue, calculated from blue SAXS data in Figure S5A. (D) Top and side views of the most-probable SAXS molecular envelopes for B1 reconstructed from SAXS data at the two conditions. Color coded the same as in A-C. The crystal structure was displayed in green color and in cartoon mode.

bioRxiv preprint doi: https://doi.org/10.64898/2026.05.01.722179; this version posted May 5, 2026. The copyright holder for this preprint (which was not certified by peer review) is the author/funder, who has granted bioRxiv a license to display the preprint in perpetuity. It is made available under aCC-BY 4.0 International license.

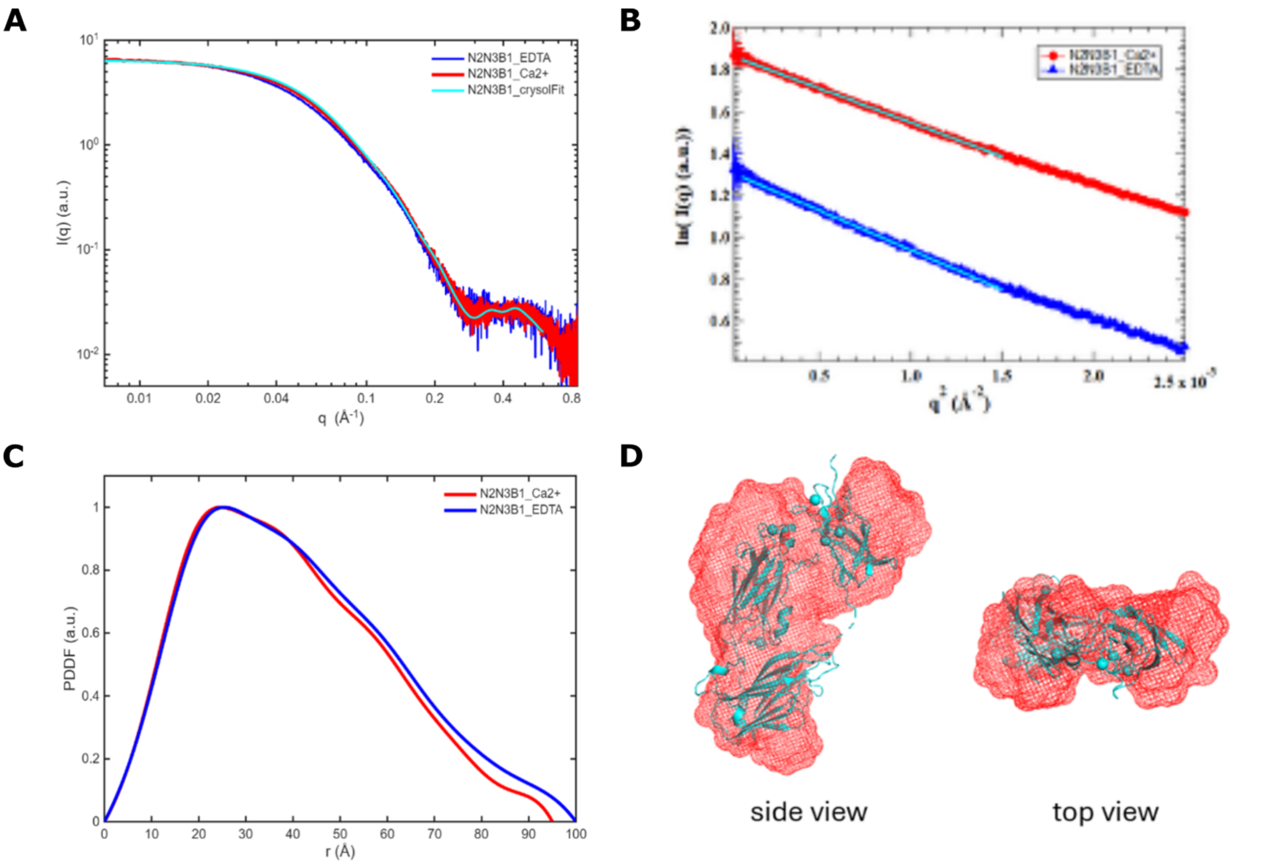

Figure S7. MD simulations on *Staphylococcus aureus* SdrD B1 domain. (**A**,**B**) Comparison of the conformational distribution of the R_g sampled for the B1 domain with and without Ca^{2+} at 300 K (**A**) and 450 K (**B**). (**C**, **D**) Comparison of the conformational distribution of root mean square deviation (RMSD) of the α carbons from the crystal structure at 300 K (**C**) and 450 K (**D**). (**E**, **F**) Representative trajectories from sample MD simulations conducted at 300 K (**E**) and 450 K (**F**). (**G**, **H**) Comparison of theoretical SAXS for the B1 domain with (**G**) and without (**H**) Ca^{2+} to the experimental SAXS data. The average MD structure represents the weighted average for the conformational distributions depicted in Figure 4C. Minor discrepancies are attributed to effects of the solvation layer and the experimental SAXS representing an ensemble average over different conformations.

bioRxiv preprint doi: https://doi.org/10.64898/2026.05.01.722179; this version posted May 5, 2026. The copyright holder for this preprint (which was not certified by peer review) is the author/funder, who has granted bioRxiv a license to display the preprint in perpetuity. It is made available under aCC-BY 4.0 International license.

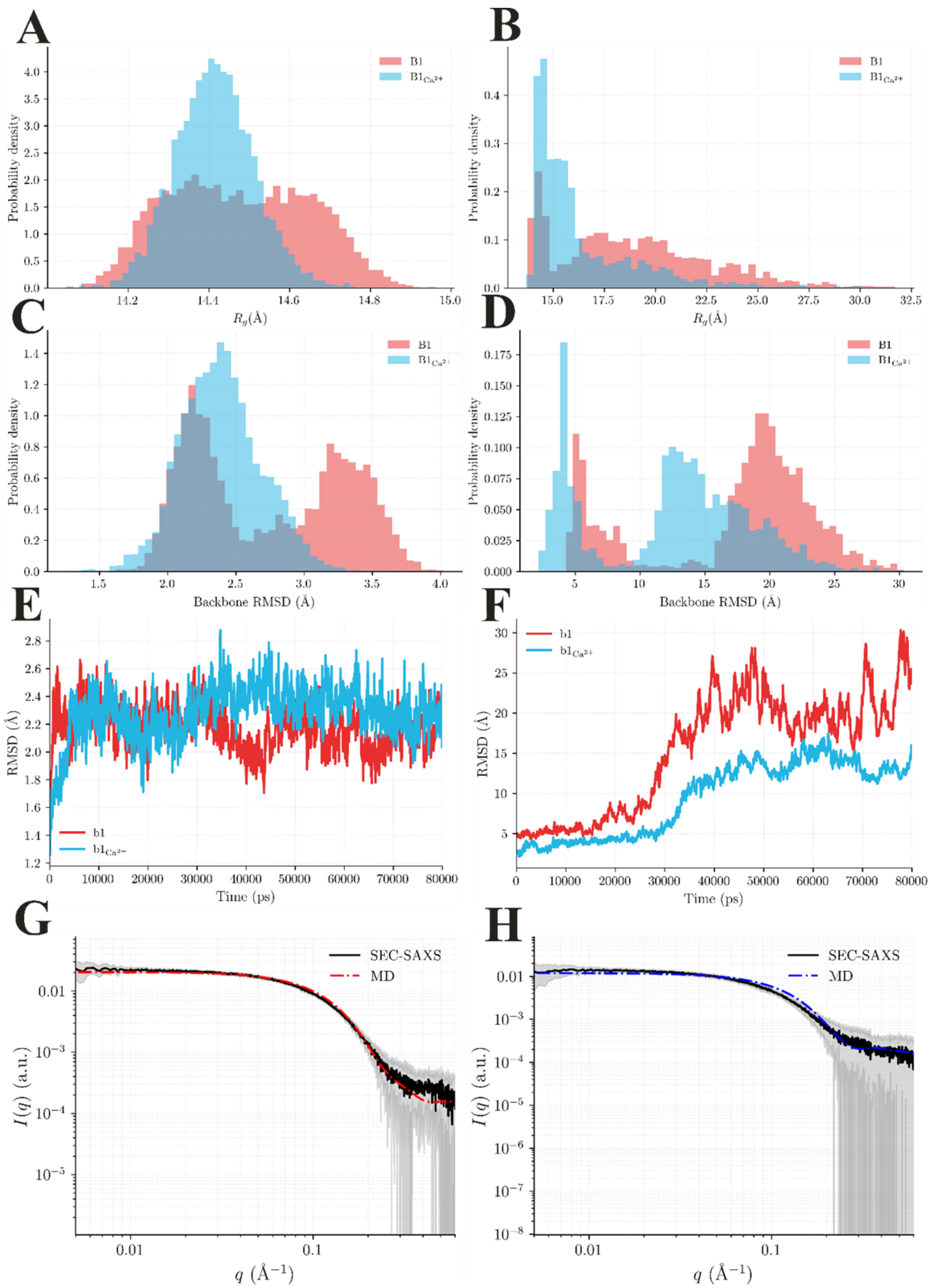

Figure S8. SAXS results for *Staphylococcus aureus* SdrD N2N3B1. (A) Superimposed SAXS profiles of N2N3B1 in protein buffer (red curve), in protein buffer with 10 mM EDTA added (blue), and fitting with the crystal structure (cyan). The goodness-of-fit (chi2) of CRYSOL fitting with the crystal structure against the red curve is 2.2. (B) Guinier plots for protein N2N3B1in the presence of Ca^{2+} (red) and EDTA (blue), respectively. Cyan lines are fittings which yield the following radius of gyration (R_g) values: 31.2±0.2 Å with Ca^{2+}; 33.6±0.2 Å with EDTA. (C) PDDF profiles for N2N3B1. Red, calculated from experimental SAXS data (red curve in Figure S7A) using GNOM; blue, calculated from blue SAXS data in Figure S7A. (D) Side and top views of the most-probable SAXS molecular envelope for N2N3B1 reconstructed for the data collected with Ca^{2+}. Color coded the same as in A-C. The N2N3B1 crystal structure was displayed in cyan color and in cartoon mode.

bioRxiv preprint doi: https://doi.org/10.64898/2026.05.01.722179; this version posted May 5, 2026. The copyright holder for this preprint (which was not certified by peer review) is the author/funder, who has granted bioRxiv a license to display the preprint in perpetuity. It is made available under aCC-BY 4.0 International license.

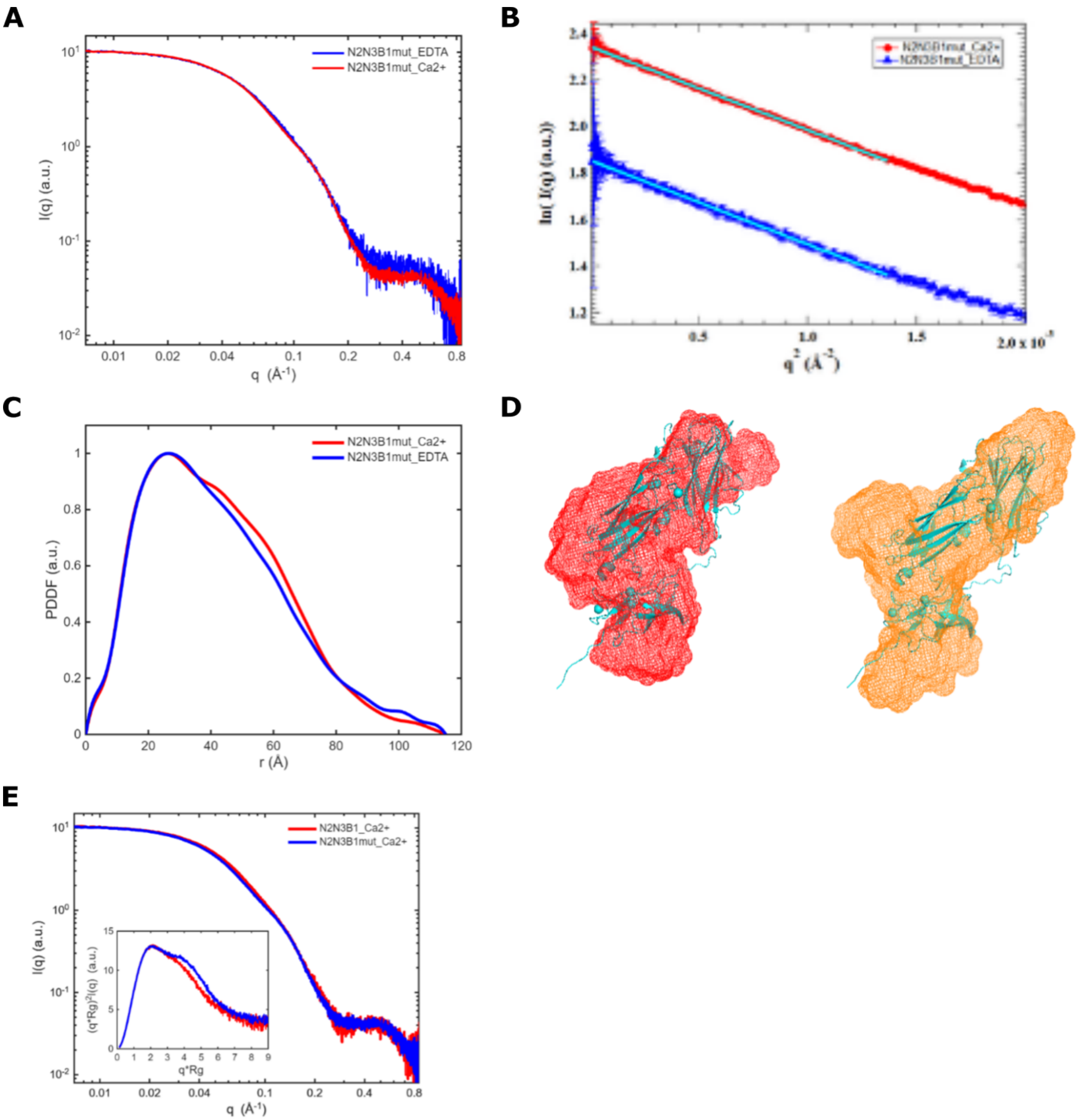

Figure S9. SAXS results for *Staphylococcus aureus* SdrD N2N3B1mut. (A) Superimposed SAXS profiles of N2N3B1mut in protein buffer (red curve), and in protein buffer with 10 mM EDTA added (blue). (B) Guinier plots for protein N2N3B1in the presence of Ca^{2+} (red) and EDTA (blue), respectively. Cyan lines are the fittings which yield radius of gyration (R_g) values: 32.8±0.2 Å with Ca^{2+}; 32.9±0.3 Å with EDTA. (C) PDDF profiles for N2N3B1mut. Red, calculated from experimental SAXS data (red curve in Figure S8A) using GNOM; blue, calculated from blue SAXS data in Figure S8A. (D) Side views of the refined SAXS molecular envelope for N2N3B1 (red) and N2N3B1mut (orange) reconstructed for data collected with Ca^{2+}. The N2N3B1 crystal structure is displayed in cyan color and in cartoon mode.

bioRxiv preprint doi: https://doi.org/10.64898/2026.05.01.722179; this version posted May 5, 2026. The copyright holder for this preprint (which was not certified by peer review) is the author/funder, who has granted bioRxiv a license to display the preprint in perpetuity. It is made available under aCC-BY 4.0 International license.

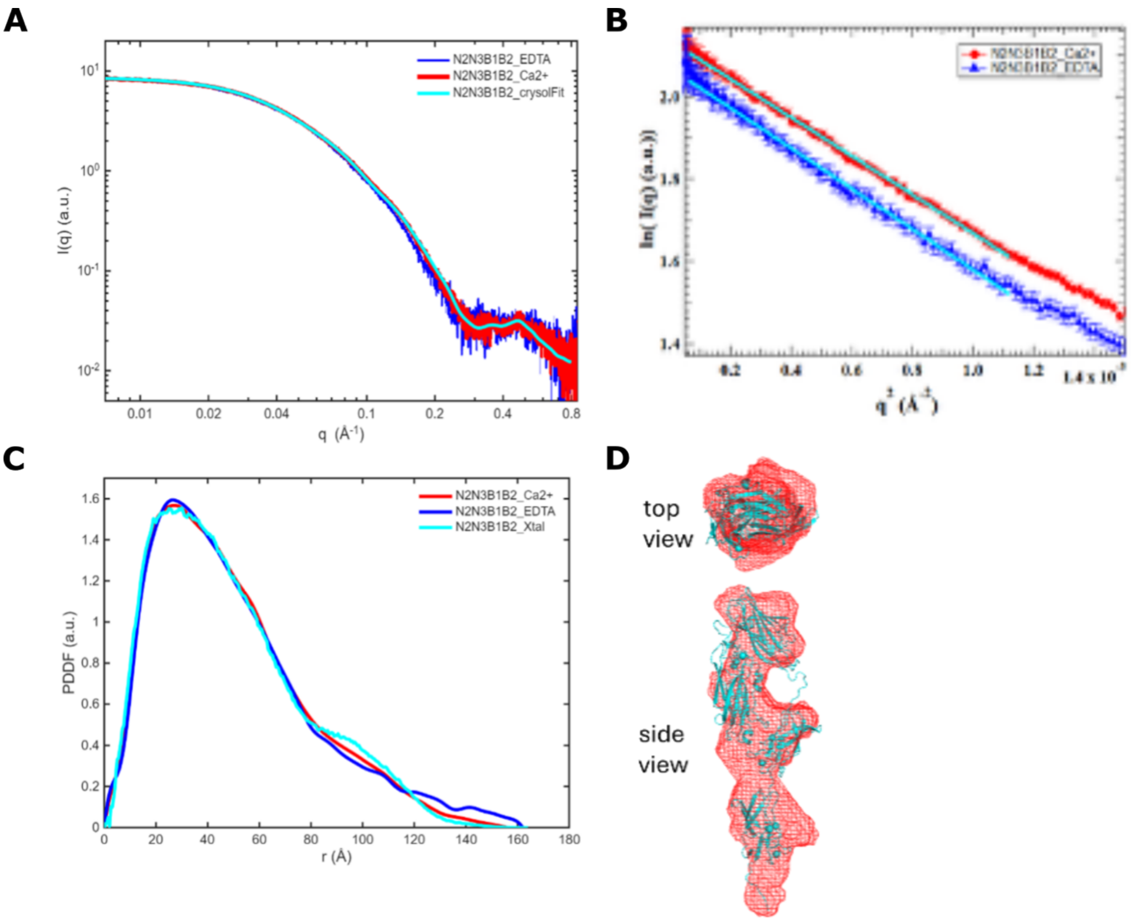

Figure S10. SAXS results for *Staphylococcus aureus* SdrD N2N3B1B2. (A) Superimposed SAXS profiles of N2N3B1B2 in protein buffer (red curve), in protein buffer with 10 mM EDTA added (blue), and fitting with the crystal structure (cyan). The goodness-of-fit (chi2) of CRYSOL fitting with the crystal structure against the red curve is 0.44. (B) Guinier plots for protein N2N3B1B2 in the presence of Ca^{2+} (red) and EDTA (blue), respectively. Cyan lines are fittings which yield the following radius of gyration (Rg) values: 37.5±0.2 Å with Ca2+; 38.3±0.3 Å with EDTA. (C) PDDF profiles for N2N3B1B2. Red, calculated from experimental SAXS data (red curve in Figure S9A) using GNOM; blue, calculated from blue SAXS data in Figure S9A. (D) Side and top views of the most-probable SAXS molecular envelope for N2N3B1B2 reconstructed for the data collected with Ca^{2+}. The N2N3B1B2 crystal structure is displayed in cyan color and in cartoon mode and superimposed with SAXS structural envelope.

bioRxiv preprint doi: https://doi.org/10.64898/2026.05.01.722179; this version posted May 5, 2026. The copyright holder for this preprint (which was not certified by peer review) is the author/funder, who has granted bioRxiv a license to display the preprint in perpetuity. It is made available under aCC-BY 4.0 International license.

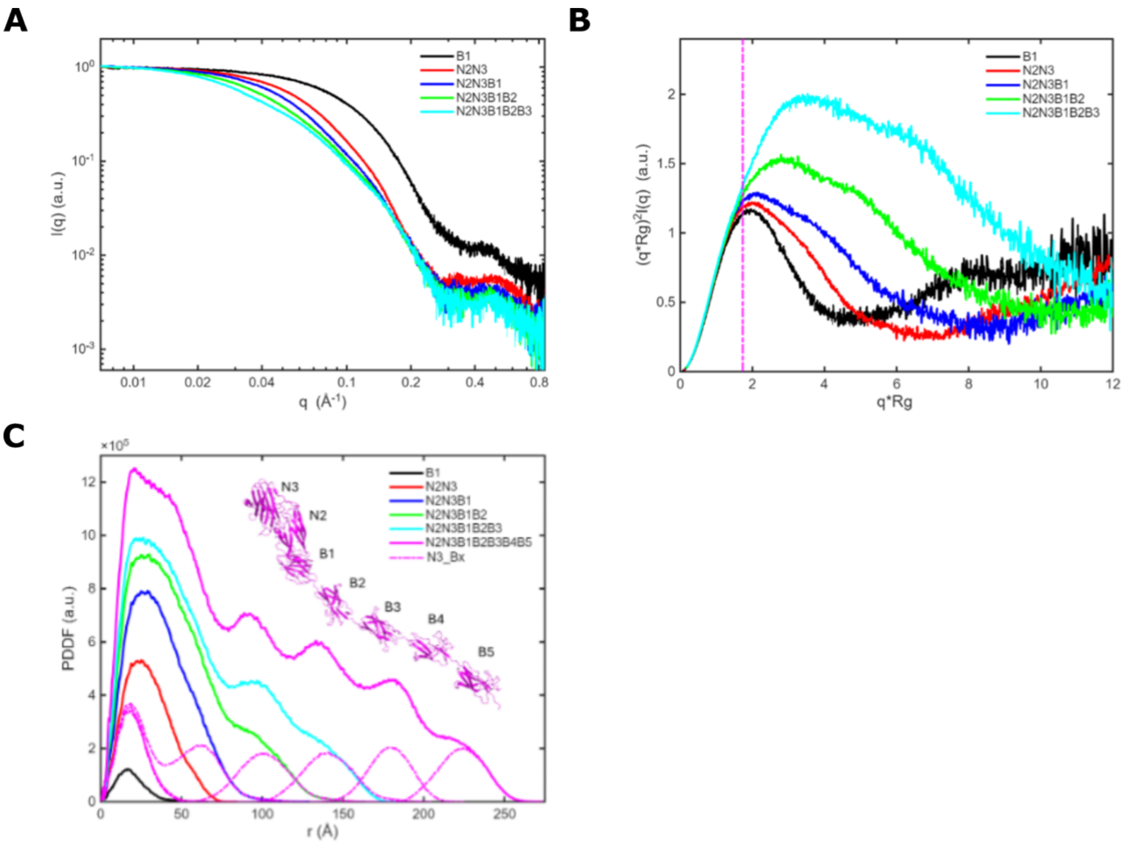

Figure S11. (A) Summary plot of normalized experimental SAXS profiles for all constructs. (B) Dimensionless Kratky plots for experimental SAXS data in (A). Colors are coded as in (A). The magenta vertical line is q*Rg=$\sqrt{3}$, where the peak of spherical particle appears. The higher the aspect ratio of length to cross-section, the further the peak position is away from $\sqrt{3}$. (C) Theoretical PDDFs of all constructs calculated from atomic structures. Among the atomic structures, B1, N2N3, N2N3B1 and N2N3B1B2 are crystal structures, and N2N3B1B2B3 and N2N3B1B2B3B4B5 are AF3 models. N2N3B1B2B3B4B5 is displayed in the inset with labels of N and B domains. The dash line profiles in magenta are five PDDFs for the ensemble structure, consisting of N3 and one individual B domains, i.e., N3_B1, N3_B2, N3_B3, N3_B4, and N3_B5, respectively. They all consist of two peaks, one short distance peak centering at ~18 Å, and one far distance peak centering from 62 to 224 Å, depending on up the location of B domain. The first peak reflects the intra-domain pair-wise distance within N3 or B domains, while the second peak describes the inter-domain pair-wise distance correlations between N3 and B domains. The peak position of the second peak reflects the inter-domain center-to-center distance.

bioRxiv preprint doi: https://doi.org/10.64898/2026.05.01.722179; this version posted May 5, 2026. The copyright holder for this preprint (which was not certified by peer review) is the author/funder, who has granted bioRxiv a license to display the preprint in perpetuity. It is made available under aCC-BY 4.0 International license.

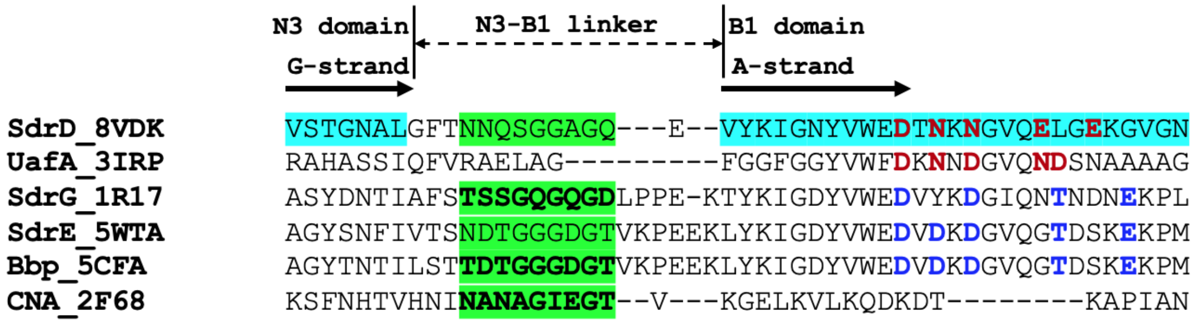

Figure S12. **Structure based sequence alignment of N3-B1 interdomain region.** Relevant Sdr and Sdr-like molecules were used for the alignment. Crystal structure PDB codes are shown after molecular names for reference. The last strand of the N3 G-strand and a part of N-terminal region of B1 domain (including A-strand) were used in the alignment. Strand assignments are based on SdrD structure. Observed (bolded) and presumed latch forming motifs within the N3-B1 linkers are highlighted in green. In the B1 domain region, observed metal ion-binding residues are in red font in SdrD and UafA. Residues in equivalent or nearly equivalent positions of metal-binding sites in SdrG, SdrE and Bbp are in blue font.
